# Bacteria-Targeted Clindamycin Loaded Polymeric Nanoparticles: Effect of Surface Charge on Nanoparticle Adhesion to MRSA, Antibacterial Activity, and Wound Healing

**DOI:** 10.3390/pharmaceutics11050236

**Published:** 2019-05-15

**Authors:** Nurhasni Hasan, Jiafu Cao, Juho Lee, Shwe Phyu Hlaing, Murtada A. Oshi, Muhammad Naeem, Min-Hyo Ki, Bok Luel Lee, Yunjin Jung, Jin-Wook Yoo

**Affiliations:** 1 College of Pharmacy, Pusan National University, Busan 46241, Korea; nurhasni.hasan@unhas.ac.id (N.H.); caojiafu1985@163.com (J.C.); jhlee2350@gmail.com (J.L.); shwephyuhlaing@gmail.com (S.P.H.); murtadaoshi98@gmail.com (M.A.O.); m.naeem@numspak.edu.pk (M.N.); brlee@pusan.ac.kr (B.L.L.); jungy@pusan.ac.kr (Y.J.); 2 Samjin Pharm. Co., LTD., Seongnam 13488, Korea; mhki@samjinpharm.co.kr

**Keywords:** MRSA-infected wound healing, clindamycin, surface charge, nanoparticles, antibacterial

## Abstract

Adhesion of nanoparticles (NPs) to the bacterial cell wall by modifying their physicochemical properties can improve the antibacterial activity of antibiotic. In this study, we prepared positively charged clindamycin-loaded poly (lactic-*co*-glycolic acid)-polyethylenimine (PLGA-PEI) nanoparticles (Cly/PPNPs) and negatively charged clindamycin-loaded PLGA NPs (Cly/PNPs) and investigated the effect of NP adhesion to bacteria on the treatment of methicillin-resistant *Staphylococcus aureus* (MRSA)-infected wounds. The Cly/PPNPs and Cly/PNPs were characterized according to particle size, polydispersity index, surface charge, and drug loading. Both Cly/PPNPs and Cly/PNPs exhibited sustained drug release over 2 days. The Cly/PPNPs bind to the MRSA surface, thereby enhancing bactericidal efficacy against MRSA compared with the Cly/PNPs. Furthermore, compared with other groups, Cly/PPNPs significantly accelerated the healing and re-epithelialization of wounds in a mouse model of a MRSA-infected wounds. We also found that both NPs are harmless to healthy fibroblast cells. Therefore, our results suggest that the Cly/PPNPs developed in this study improve the efficacy of clindamycin for the treatment of MRSA-infected wounds.

## 1. Introduction

Bacterial infection can alter and affect the process of wound healing [1]. The degree of disturbance in the orderly scheme of healing is affected by high tissue bacterial levels, the type of bacteria, host immune response, and the bacterial products (e.g., metalloproteinases and endotoxins) [2,3]. Cases of methicillin-resistant *Staphylococcus aureus* (MRSA) infection of cutaneous wound have escalated significantly in the past decade. The MRSA bacterium itself has emerged as a community-acquired (CA) infection that causes hospitalization globally [4,5,6,7,8]. The major problem resulting from MRSA infection of wound is its spread to other organs in the body, developing into a deep and invasive infection, resulting in systemic diffusion (bacteremia) and significant morbidity and mortality worldwide [9,10]. The associated case fatality ratio is 20.3%, and the mortality rate is 3.4 per 100,000 individuals per year [11,12]. MRSA infection can also be contagious. Although highly active antimicrobial agents are available, the treatment of MRSA-infected wounds remains challenging due to the lack of an innovative drug-carrier system. Therefore, MRSA-infected wounds need proper treatment and urgent medical attention.

Clindamycin is an FDA-approved drug used to treat MRSA bacterial infections. Unlike other antimicrobial agents such as doxycycline, minocycline, trimethoprim, sulfamethoxazole, rifampin, and linezolid, its use has been less restrained by safety considerations and precautions (e.g., drug–drug interactions, inadvisable amid pregnancy or children below the age of 8, and in association with myelosuppression, neuropathy, and lactic acidosis over lengthened therapy) [13,14]. A semisynthetic derivative of lincomycin, clindamycin acts by inhibiting ribosomal translocation or protein synthesis [15]. The treatment of MRSA-infected wounds with clindamycin may proceed via tablet/suspension (oral), IV (systemic), and gel/cream (topical application) form [16,17], all of which have several drawbacks, including adverse side effects, the uneven distribution of antibiotic to bacteria, and the possibility of the emergence of bacterial resistance [18]. The conventional approach toward treatment requires administration of the drug daily for up to 7 days, which may lead to patient incompliance. To overcome these problems, a means to deliver clindamycin to the bacteria in a sustained manner is highly needed.

In recent years, the topical delivery of antibiotics through nanoparticle (NP) drug carries have shown potential efficacy in treating bacterial infections [19,20]. NPs are a promising drug delivery system that enables controlled and sustained release of antibiotics. Their subcellular size and amenability to surface charge modification enable them to interact with the biological milieu and improve drug efficacy in vivo [21,22,23,24,25]. NPs also provides several benefits such as (1) enhanced antibiotic treatment results with fewer side effects, (2) diminished probability of antibiotic resistance arising from ineffective drug dosing, and (3) high sustained local drug concentrations [26,27,28]. Nanocarriers of clindamycin include solid lipid nanoparticles (SLNs) [29], calcium phosphate NPs (CAP NPs) [18], poly(d,l-lactide-*co*-glycolide)/hydroxyapatite (PLGA/HA) core-shell nanospheres [30], and clindamycin/silver NPs [31]. Radovic-Moreno et al. suggested that the intracellular delivery of antibiotic through NP carriers is important because many types of bacterial colonies produce an acidic environment that lowers the efficacy of the pure form of antibiotics [22]. They emphasized the importance of surface charge-switching polymeric NPs for the bacterial cell wall-targeted delivery of vancomycin. Their results are consistent with our previous findings [32] demonstrating that modified NPs are a key factor in improving the effectiveness of nitric oxide as an antibacterial agent and relied on the fact that most pathogenic bacteria have negatively charged cell walls. In addition, the bacterial cell wall is the outermost layer and the most accessible part of bacteria; its integrity is pivotal for bacterial survival.

In the present work, we developed bacteria-targeted, clindamycin-loaded polymeric NPs for the effective treatment of MRSA-infected wounds. PLGA is FDA-approved and has long been used for controlled release drug delivery. It offers the flexibility to fine-tune its surface properties [33,34,35]. Polyethylenimine (PEI) was used as the cationic-donor polymer to prepare positively charged, clindamycin-loaded PLGA NPs (Cly/PPNPs). Negatively charged, clindamycin-loaded PLGA NPs (Cly/PNPs) were also prepared as counterpart particles. After evaluating their physicochemical properties, NPs were tested for in vitro drug release, in vitro bacterial adhesion, and in vitro and in vivo bactericidal effects. In vivo wound healing activity was evaluated in an ICR mouse model of MRSA-infected wound.

## 2. Materials and Methods

### 2.1. Materials

PLGA (50:50 DLG 5E) was purchased from Lakeshore Biomaterials (Birmingham, AL, USA). Clindamycin HCL was a generous gift from Samjin Pharmaceutical Company. PEI (*M*_W_ 1.8 kDa), poly(vinyl alcohol) (PVA), 2,2,2-tribromoethanol and *tert*-amyl alcohol (2-methyl-2-butanol) (Avertin anesthesia component), Mayer’s hematoxylin, eosin-Y disodium salt, Nile red, tetrazolium dye 3-(4,5-di-methylthiazol-2-yl)-2,5-diphenyltetrazolium bromide (MTT), and dimethylsulfoxide (DMSO) were purchased from Sigma-Aldrich (St. Louis, MO, USA). Bacto^TM^ tryptic soy broth (TSB) medium was purchased from BD Biosciences (Sparks, MD, USA). The LIVE/DEAD^®^ Baclight^TM^ bacterial viability kit (Molecular Probes) was purchased from Life Technologies (Eugene, OR, USA). The Roswell Park Memorial Institute (RPMI) 1640 medium, trypsin, fetal bovine serum (FBS), and penicillin-streptomycin were purchased from Hyclone, Thermo Fisher Scientific Inc. (Waltham, MA, USA). Phosphate buffered saline (PBS; 20×) was purchased from Biosesang (Seoul, Korea). All other reagents and solvents used were of the highest analytical grade.

### 2.2. NP Preparation

The PLGA-PEI NPs (PPNPs), Cly/PPNPs and Cly/PNPs were prepared using an oil-in-water (o/w) emulsification solvent evaporation method [36,37]. Briefly, PLGA (200 mg) was mixed with 20 mg of either PEI or clindamycin base and dissolved in 10 mL dichloromethane. For fluorescence labeling, Nile red (0.5 mg) was added to the polymer solution. The organic phase was poured into a 30 mL solution of 1% PVA and then was emulsified using a high-speed homogenizer (Ultra Turrax T-10, IKA Werke, GmbH & Co.KG, Staufen im Breisgau, Germany) at 14,500 rpm for 2 min in an ice bath, followed by probe sonication (KFS-300N ultrasonic, Korea Process Technology, Seoul, Korea) at a power of 150 W for 3 min in an ice bath. The resultant emulsion was then stirred for 4 h at 400 rpm in a fume hood. After the residual solvent was removed, NPs were collected by centrifugation at 20,000× *g* for 30 min and then washed three times with distilled water (DW).

### 2.3. Characterization of NP

#### 2.3.1. Scanning Electron Microscopy (SEM)

The morphology of PPNPs, Cly/PNPs, and Cly/PPNPs was analyzed by field emission electron microscopy using the EBSD system (FE-SEM, Supra 40VP, Carl Zeiss AG, Oberkochen, Germany). The NPs were mounted on carbon tape followed by platinum coating for 2 min under vacuum. The morphology of NPs was then observed with the FE-SEM at acceleration voltages of 1–5 kV. The particles sizes (*n* = 300) were measured using ImageJ software (version 1.52i, National Institutes of Health, Bethesda, MA, USA, 2018).

#### 2.3.2. Particle Size and Surface Charge Analysis

The mean particle size, polydispersity index (PDI), and surface charge were measured using a Zetasizer Nano ZS90 (Malvern Instruments Korea, Seoul, Korea). The PDI values and mean particle size of the NPs were determined by dynamic light scattering analysis. All the measurements were measured at a set angle of 90° and diluted in Milli-Q water at 25 °C with three replications. Disposable folded capillary cells DTS 1070 were used to measure the zeta potential. The NP particle size distribution was described using the cumulants mean (z-average) ± standard deviation (SD) and the PDI.

#### 2.3.3. Drug Loading

High-performance liquid chromatography (HPLC, Shimadzu, Kyoto, Japan) analysis was used to determine the concentration of clindamycin loaded in NPs. The HPLC system was fitted out with an SPD-20A Prominence UV/Vis detector, an LC-20AT liquid chromatograph, a CT-20A Prominence column oven, a DGU-20ASR degassing unit and an SIL-20 Prominence autosampler. A VDSpher^®^ PUR 100 C18-M-SE column (5 μm, 250 × 4.6 mm, VDS Optilab, Berlin, Germany) was used. A clindamycin standard stock solution was prepared by dissolving clindamycin base in the mobile phase solution consisting of acetonitrile and phosphate buffer (pH 3.0) (52:48, *v*/*v*). The mixture was filtered through a 0.45 μm nylon membrane and pumped at a flow rate of 1.0 mL/min. Samples were injected at a volume of 20 μL, and the elution was monitored at 210 nm.

Specific amounts of Cly/PNPs and Cly/PPNPs were dissolved in acetonitrile and sonicated for 4 h and then centrifuged at 20,000× *g* for 15 min. Supernatants were diluted with the mobile phase to a suitable dilution, and each sample (100 μL) was transferred to the HPLC autosampler cell. The same chromatographic conditions as described above were used. The peak area and the clindamycin concentration were linearly correlated (*R*^2^ = 0.9993) within the concentration range of 0.004 to 1 mg/mL. All samples were prepared in triplicate.

### 2.4. In Vitro Drug Release

The release of clindamycin from Cly/PNPs and Cly/PPNPs was evaluated in PBS (pH 7.4) at 37 °C. NPs (100 mg) were dispersed in 5 mL PBS and then incubated in a shaking water bath at 100 rpm. At a preset time intervals, the pellet and supernatant were separated by centrifugation at 20,000× *g* for 15 min. One hundred microliters of supernatant was withdrawn, and the volumes were made up with fresh buffer solution. The supernatant containing clindamycin released from the NPs were analyzed using HPLC as described above.

### 2.5. NP Adhesion to the Bacteria

To evaluate the adhesion of the NPs to the bacteria, MRSA was incubated with Nile red-labeled NPs (5 mg/mL) in the TSB medium for 1 h at 37 °C with mild shaking. The bacterial suspensions were centrifuged at 8000× *g* and pellets were rinsed (3× TSB) to wash out the unbound NPs. Bound NPs were detached using a bath sonicator for 15 min to prove strong binding capability. The bacterial suspensions were then washed with 1 mL of 0.85% NaCl (2×) by centrifugation at 8000× *g* to remove media residue. After staining with with Syto-9 dye, the bacteria cells were and observed by a confocal microscope (Fluoview FV10i, Olympus Corporation, Tokyo, Japan). To observe binding using with FE-SEM, the final bacterial suspensions were fixed with 2.5% glutaraldehyde in PBS (pH 7.4) for 30 min at 25 °C. The bacterial suspensions were then rinsed with PBS and then dehydrated with a series of ethanol solution with concentration of 50%, 60%, 70%, 80%, and 90% for 15 min, and 100% ethanol for 1 h. Samples were then placed on carbon tape and air dried at room temperature in a desiccator connected to a vacuum. Samples were then coated with platinum for 2 min under vacuum and viewed by FE-SEM (Supra 40VP, Carl Zeiss AG, Oberkochen, Germany) at an accelerating voltage of 1–5 kV.

### 2.6. In Vitro Antibacterial Study

To evaluate the antibacterial activity of NPs, bacterial viability was assayed by enumerating the number of colony-forming units (CFU) and confocal microscopy was used to visualize live and dead bacteria after incubation with NPs. The bacterial strain MRSA (USA300) [38] was inoculated on TSB and grown overnight at 37 °C to the mid-exponential phase. The resulting bacterial suspension was centrifuged at 8000× *g* for 15 min and the concentration was re-adjusted with sterile PBS. Bacterial suspension was added into TSB, followed by NPs suspension (PPNPs, Cly/PPNPs, and Cly/PNPs) to final NP concentrations of 0.1, 0.3, and 0.5 mg/mL in 12-well plates. A tube containing bacteria in sterile PBS was used as a control. All the samples were incubated at 37 °C for 12 and 24 h in a shaking incubator, then centrifuged at 8000× *g* and washed twice with 0.85% NaCl. To observe the effect of binding on the antibacterial activity of the NPs, all the samples were first incubated with an MRSA suspension (equal to 0.5 McFarland standards, 1.5 × 10^8^ CFU/mL) for 1 h at 37 °C with mild shaking. The suspensions were then centrifuged at 8000× *g* and pellets were washed two times with fresh medium to remove the unbound NPs; this was followed by detachment using a bath sonicator for 15 min and fresh media was then added, and incubation was continued for up to 24 h.

To measure bacterial viability via CFU counts, the bacterial suspensions were diluted from 10^1^ to 10^8^ using PBS. A 200 µL aliquot of each dilution was plated on TSB agar and incubated at 37 °C overnight. The number of colonies was counted to calculate the number of viable bacteria at the time of plating. To discriminate between live and dead bacteria, LIVE/DEAD^®^ Baclight^TM^ bacterial viability kit reagents were used corresponding to the manufacturer’s protocol. The images were viewed with confocal microscope (Fluoview FV10i, Olympus Corporation, Tokyo, Japan). Bacteria stained with Syto-9 (green color) at excitation (ex)/emission (em) wavelengths of 539/570–620 nm indicates live cell and those stained with propidium iodide (PI, red color) at ex/em 470/490–540 nm indicates dead cells.

### 2.7. In Vitro Cytotoxicity Study

L929 mouse fibroblast cells were obtained from the Korean Cell Line Bank (Seoul, Korea). Cells were grown to subconfluency in RPMI with 10% (*v*/*v*) FBS supplemented with antibiotics (100 µg/mL streptomycin sulfate and 100 IU/mL penicillin G sodium). The cells were trypsinized, suspended in media at concentration of 5 × 10^4^ cells per well, and then plated onto 96-well plates. After 48 h incubation, the medium from each well was removed. Fresh media containing NPs were added at concentration of 0.1, 0.3, and 0.5 mg/mL (100 μL). After 24 h incubation, a standard MTT viability assay was performed. MTT solution in sterile PBS was mixed to each well and the incubation was continued for 2 h. After that, all solution was then removed from the well and 150 µL DMSO was added to solubilize the crystals. The absorbance measured at 540 nm was comparable to the concentration of viable cells in every well. The untreated cells were used as control. The viability of fibroblast cells in the presence of control and clindamycin loaded polymeric NPs is reported relative to the fibroblast viability not exposed to clindamycin NPs. The fibroblast viability was determined using equation below:

(1) $$\mathrm{Cell}\;\mathrm{viability}\;\left(\%\right)=\frac{Absorbance\;\left(treated\;cells\right)}{Absorbance\;\left(control\;cells\right)}\times 100$$

### 2.8. In Vivo Wound Healing Assay

All animal experiments were carried out in accordance with the regulations of Korean Legislation on animal studies and were approved by the Ethical Scientific Committee from the Pusan National University, Busan, Korea on 19 October 2018 as stated in the document PNU-2018-1800. Male ICR mice (7–8 weeks, Samtako Bio Korea, Osan-si, Korea) were used. The mice were first anesthetized intraperitoneal using Avertin. The dorsal hair was trimmed with an electric razor, and the skin was cut out with an 8 mm biopsy punch to create full-thickness wounds. Then, a suspension containing 1.0 × 10^8^ MRSA (USA300) was inoculated to induce infection. The freeze-dried PPNPs (0.5 mg/mL), Cly/PNPs (0.49 mg/mL NPs~7 μg clindamycin), and Cly/PPNPs (0.53 mg/mL NPs~7 μg clindamycin) were topically applied from day 2 post injury. Each wound was covered with Tegaderm^®^ and sterile gauze. Untreated mice were used as control. The gauze was replaced every 2 days. Photographs of the wounds were analyzed by ImageJ software (version 1.52i, National Institutes of Health, Bethesda, MA, USA, 2018) to measure the wound size reduction using the following equation:

(2) $$\mathrm{Wound}\;\mathrm{size}\;\mathrm{reduction}\;\left(\%\right)=\frac{W_{t}}{W_{0}}\times 100$$

where, *W*_0_ is the area of the wound at starting time 0, and *W_t_* is the area of the wound at time *t*.

### 2.9. Histological Processing

On the last day of the experiment, the cross sectional full-thickness skin specimens were excised, fixed in 10% formaldehyde for 24 h, and blocked with paraffin. Five-micron-thick vertical sections were fixed to glass slides and processed with hematoxylin and eosin (H&E) stain to observe morphology. The slides were analyzed by light microscopy (Olympus BX53, Olympus Corporation, Tokyo, Japan), and images were digitally captured at a resolution of 1360 × 1024 pixel with an Olympus DP70 digital camera.

### 2.10. Reduction of Wound Bacterial Burden

The bacterial burden (bacterial viability) on the wound was monitored from 2 to 8 days post injury. At day 2 post injury and MRSA inoculation, a sterile swab with PBS was applied to sample the biofilm growing on the wound and plated on TSB agar for a qualitative examination. Following examination of the wound at days 4 and 8 after MRSA inoculation, wound skin tissues were homogenized and diluted in sterile PBS. Two hundred microliters of each dilution was plated on TSB agar and incubated at 37 °C overnight. The number of colonies was counted and used to calculate the number of viable bacteria at the time of plating.

### 2.11. Statistical Analysis

Statistical analysis was executed using one-way analysis of variance (ANOVA) and Bonferroni multiple comparison tests or through an unpaired t-test in GraphPad Prism 5.03 (GraphPad Software, La Jolla, CA, USA, 2009). In cases of significant deviations from the t-test, the nonparametric Mann–Whitney U tests were conducted to compare the distributions of two unpaired groups. A value of *p* < 0.05 was regarded statistically significant. The data are shown as the means ± SD.

## 3. Results

### 3.1. Characterization of NPs

**Figure 1:**
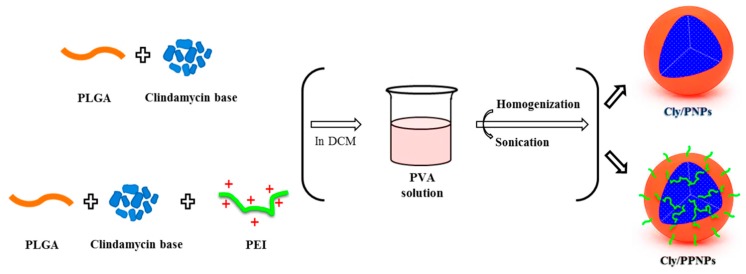
Fabrication of surface charged clindamycin NPs (Cly/PNPs and Cly/PPNPs).

**Figure 2:**
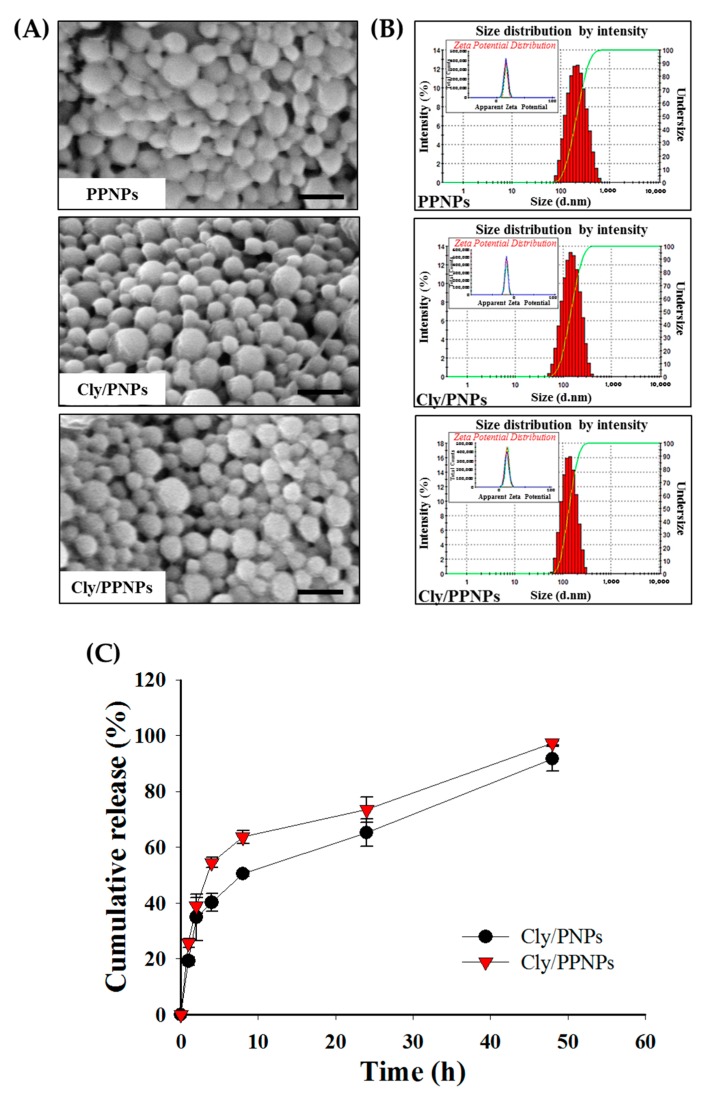
Characterization of NPs. (**A**) Scanning electron microscope (SEM) images of PPNPs, Cly/PNPs, and Cly/PPNPs; bars represent 300 nm. (**B**) Size distribution of PPNPs, Cly/PNPs, and Cly/PPNPs by zetasizer nano series ZS90; insets represent zeta potential measurement. (**C**) In vitro release profile of Cly/PNPs, and Cly/PPNPs. All samples were placed in phosphate buffered saline (PBS) pH 7.4 at 37 °C; data are means ± SD; *n* = 3.

The NPs (PPNPs, Cly/PNPs and Cly/PPNPs) were prepared by an (o/w) emulsification solvent evaporation method (Figure 1), producing NPs with physicochemical properties (morphology, size, PDI, zeta potential, and drug loading) shown in Table 1. SEM images of the prepared NPs reveal a spherical morphology with uniform particle size (Figure 2A). The average sizes of the PPNPs, Cly/PNPs and Cly/PPNPs were 193 ± 38 nm, 132 ± 41 nm, and 126 ± 33 nm, respectively. The particle size distributions (Figure 2B) and PDI values reveal a moderate polydisperse distribution type. The zeta potential measurements reveal that the PPNPs and Cly/PPNPs are positively charged (+17 ± 0.5 mV and +13 ± 0.6 mV, respectively), whereas the Cly/PNPs are negatively charged with a zeta potential value of −16 ± 0.2 mV. The blank PLGA NPs (PNPs) yielded a similar result as the PPNPs (data were not shown). The drug loading levels of Cly/PNPs and Cly/PPNPs were 1.43 ± 0.45% *w*/*w* and 1.31 ± 0.26% *w*/*w*, respectively.

### 3.2. In Vitro Drug Release

The in vitro release of clindamycin from PLGA NPs is illustrated in Figure 2C. Clindamycin was released from Cly/PNPs and Cly/PPNPs in a biphasic pattern, with an initial fast release within the first 8 h followed by a gradual and sustained release over 2 days. In the first 8 h, clindamycin was released slightly faster from Cly/PPNPs compared with Cly/PNPs. As much as 54.5% of the clindamycin was released from the Cly/PPHPs within the first 4 h, followed by 63.6% at 8 h, and 97.3% at 2 days. On the other hand, clindamycin was released from Cly/PNPs at slightly slower rates, achieving 40.2% release at 4 h, and 50.5% release at 8 h, finally reaching 91.7% release in 2 days.

### 3.3. Adhesion of NPs to MRSA

**Figure 3:**
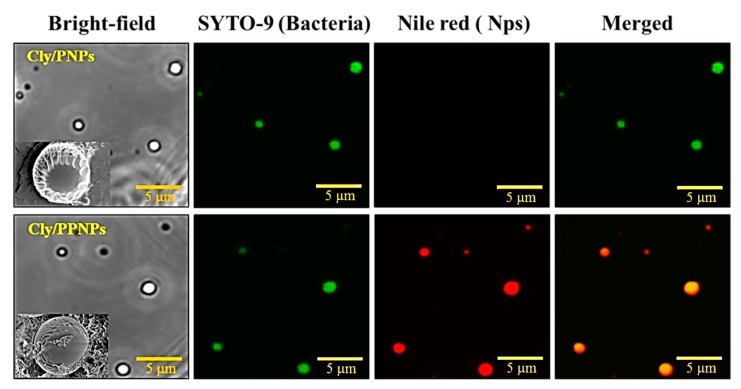
Adhesion of Cly/PNPs and Cly/PPNPs to bacteria. NPs were incubated with bacteria for 1 h and images were obtained using a confocal microscope. Bacterial membrane (green) is stained with Syto-9, and NPs (red) are labeled with Nile red. Inset images in figures show SEM images of NPs bound to bacteria.

The adhesion of NPs to the MRSA cell wall was evaluated by confocal microscopy. In Figure 3, the green and red colors represent Syto-9-labeled bacteria and Nile red-labeled NPs, respectively. The positively charged NPs (Cly/PPNPs) attached to the bacterial wall of MRSA are seen as yellow in merged images. However, the negatively charged clindamycin NPs (Cly/PNPs) showed no binding activity. Notably, the adhesion of the NPs to the bacteria was preserved even after sonication (visualized in confocal images), which is indicative of strong binding. The electrostatic adhesion of Cly/PPNPs can also be observed in the SEM images. The lower inset images in Figure 3 shows the attachment of Cly/PPNPs on the surface of bacteria, whereas the upper inset figure shows bacteria free of Cly/PNPs. This confirms that the attachment of NP to bacteria surface is an electrostatic interaction

### 3.4. In Vitro Antibacterial Activity of NP

**Figure 4:**
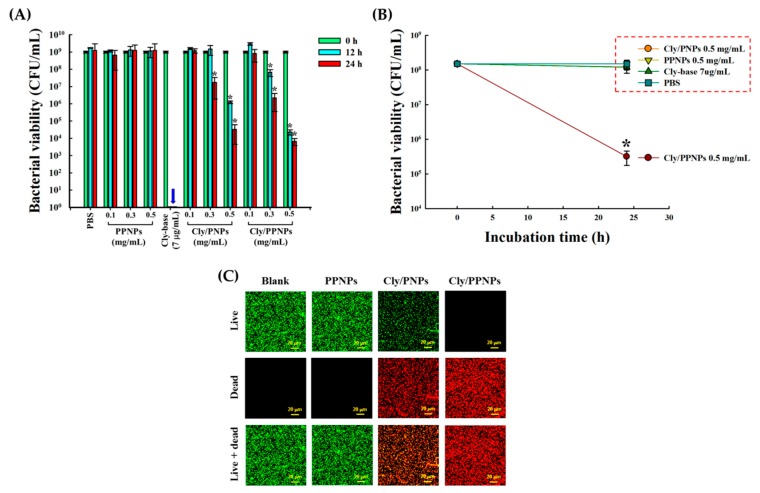
Antibacterial activity of PPNPs, Cly/PNPs, and Cly/PPNPs against MRSA. (**A**) The mean number of CFUs ± SD (*n* = 3). The blue arrow at Cly-base shows no viable bacteria (0 CFU/mL). (**B**) The effect of binding on bacterial viability after treatment with or without Cly-base, PPNPs, Cly/PNPs, and Cly/PPNPs, followed by washing. The red box indicates a group of samples that have no antibacterial activity. (**C**) Confocal microscopy images after 24 h of treatment with NPs at 0.5 mg/mL. Syto-9 fluorescence (green) represents the intact membrane of live bacteria, PI fluorescence (red) represents membrane destruction and cell death. Blank is the control group (PBS alone). Bars represent 20 μm.

The in vitro antibacterial activities of the PPNPs, Cly/PNPs, and Cly/PPNPs against MRSA were evaluated based on CFU counts (Figure 4A,B) and by confocal microscopy to visualize live and dead bacteria (Figure 4C). The results shown inFigure 4A,B highlight the importance of bacterial adhesion on antibacterial activity. No antibacterial activity against MRSA was observed with blank NPs (PPNPs) at different concentrations (0.1, 0.3, and 0.5 mg/mL) and incubation times (12 and 24 h). In sharp contrast, both drug-loaded NPs (Cly/PNPs and Cly/PPNPs) showed significant antibacterial activities in a concentration- and time-dependent manner (Figure 4A). At the lowest NP concentration (0.1 mg/mL Cly/PNPs and Cly/PPNPs), bacterial viability remained unchanged within the first 12 h of incubation but tended to decrease at 24 h. At 0.3 mg/mL Cly/PPNPs, bacterial viability was reduced by 1-log (90% killing) within 12 h of incubation, whereas Cly/PNPs had no antibacterial effect. However, by 24 h of incubation, both Cly/PNPs and Cly/PPNPs caused >2-log reduction of bacterial viability (~99% of killing). At 0.5 mg/mL Cly/PNPs caused 3-log reduction of bacterial viability (99.9% of killing) within 12 h of incubation, which increased to >4-log reduction (99.99% of killing) after 24 h of incubation. Cly/PPNPs (0.5 mg/mL) exerted greater antibacterial effect than Cly/PNPs, causing a >4-log reduction (99.99% of killing) in the first 12 h of incubation, increasing to a 5-log reduction (99.999% of killing) at 24 h of incubation. The effect of positive charge on the adhesion of NPs to the bacterial cell wall is shown in Figure 4B. Different concentration (0.1, 0.3, and 0.5 mg/mL) of PPNPs, Cly/PNPs, and Cly/PPNPs were incubated in bacterial suspensions for 24 h, followed by a washing procedure to remove unattached NPs. Cly/PPNPs at 0.5 mg/mL caused a significant 2-log reduction of bacterial viability (~99% of killing) as compared to drug-free solution, blank NPs and Cly/PNPs. Clindamycin solution showed a higher level of antibacterial activity compared with drug-loaded NPs (Figure 4A); however, when cells were washed, it exerted no antibacterial activity (Figure 4B). Thus, the ability to bind to the bacterial cell is significant, because the drug-free solution and unattached NPs can be washed away. Figure 4C shows the viability of the bacteria as visualized by a confocal microscope after live-dead staining. The green (Syto-9) and red (PI) fluorescence represent live and dead bacteria, respectively. The images show bacteria incubated with 0.5 mg/mL PPNPs, Cly/PNPs, and Cly/PPNPs for 24 h without washing. The blank NPs (PPNPs) were associated with a high number of live cells, with no dead cells detected. In contrast, Cly/PNPs and Cly/PPNPs were associated with a relatively lower number of live cells compared with dead cells. The Cly/PPNPs exerted a greater effect than Cly/PNPs as seen by the undetectable levels of live cells and the high number of dead cells in this treatment. This result agrees with the result of viable cell counts that show 0.5 mg/mL Cly/PPNPs caused a 5-log reduction of bacterial viability (CFUs) (99.999% of killing).

### 3.5. In Vitro Cytotoxicity Study

**Figure 5:**
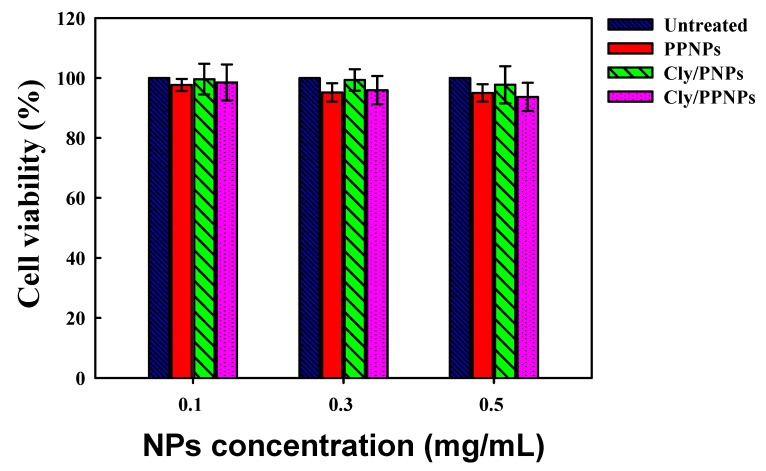
Viability (%) of L929 mouse fibroblast cells following 24 h of exposure to various concentrations of NPs (*n* = 8).

The in vitro cytotoxicity of PPNPs, Cly/PNPs, and Cly/PPNPs to L929 mouse fibroblast cells are shown in Figure 5. The results show that all NPs exert no significant cytotoxicity (>93% viability) to L929 fibroblast cells, regardless of concentration.

### 3.6. In Vivo Wound Healing Assay

**Figure 6:**
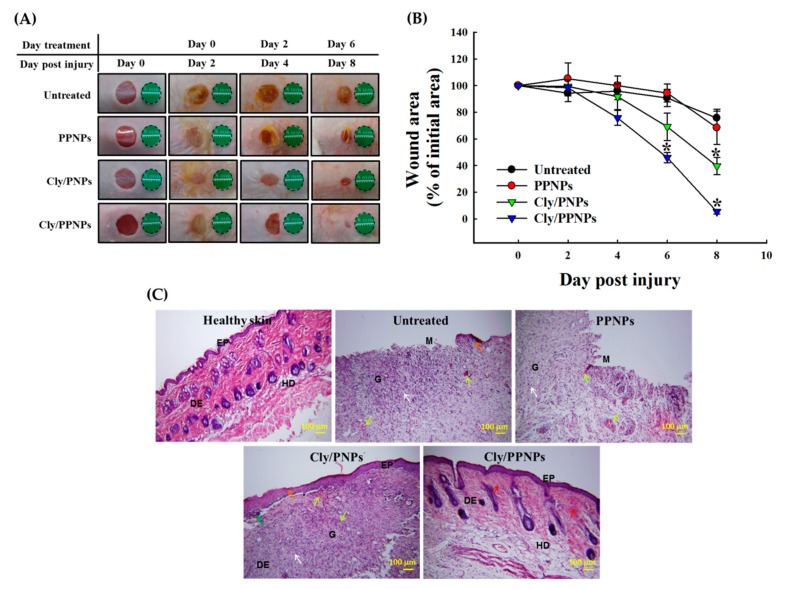
Wound healing assay in mice. (**A**) Representative photographs of methicillin-resistant *Staphylococcus aureus* (MRSA)-infected wounds of ICR mice treated with or without PPNPs, Cly/PNPs, and Cly/PPNPs. (**B**) Area reduction (%) profiles of the wounds. Values are mean ± SD, *n* = 10 different wounds, “*” indicates *p* < 0.05 compared with untreated group. (**C**) Histological analysis (H&E staining) of MRSA-infected wounds of ICR mice at day 8. Scale bar = 100 μm. Ep = epidermal, DE = dermal junction, HD = hypodermis, G = granulation tissue, and M = wound matrix. The orange arrows indicate early epithelialization. The green arrow shows skin crust, red arrows indicate fibroblast cells, white arrows denote mononuclear inflammatory cells and yellow arrows show neovascularization.

The in vivo wound healing assay was carried out to investigate whether Cly/PNPs and Cly/PPNPs can accelerate the repair of MRSA-infected wounds (Figure 6). Visually, Cly/PNP and Cly/PPNP treatments caused reductions in the wound bacterial burden, followed by accelerated wound healing, whereas untreated and blank NPs (PPNPs) did not affect the bacterial burden or the wound size (Figure 6A). In Cly/PPNPs-treated groups, the in vivo reduction of bacterial burden was observed at day 4 post injury. Wound closure (% of initial area) at day 8 post injury in response to Cly/PNPs and Cly/PPNPs treatments were 60.4% (*p* < 0.05) and 94% (*p* < 0.05), respectively (Figure 6B).

### 3.7. Histological Analysis

The histological examination of skin specimens following H&E staining shows the skin layer morphology at day 8 post injury. Unlike the untreated and PPNPs groups, the Cly/PNPs- and Cly/PPNPs-treated mice show a skin morphology similar to normal skin (Figure 6C). H&E staining of the untreated and PPNPs group samples show open wound and early epithelialization, ulceration, and plenty of mononuclear inflammatory cells infiltrated through the dermal layer. In contrast, Cly/PNPs and Cly/PPNPs group samples show increased numbers of fibroblast-like cells and decreased numbers of mononuclear inflammatory cells and healed skin structures that appear similar to normal healthy skin.

### 3.8. Reduction of Wound Bacterial Burden

**Figure 7:**
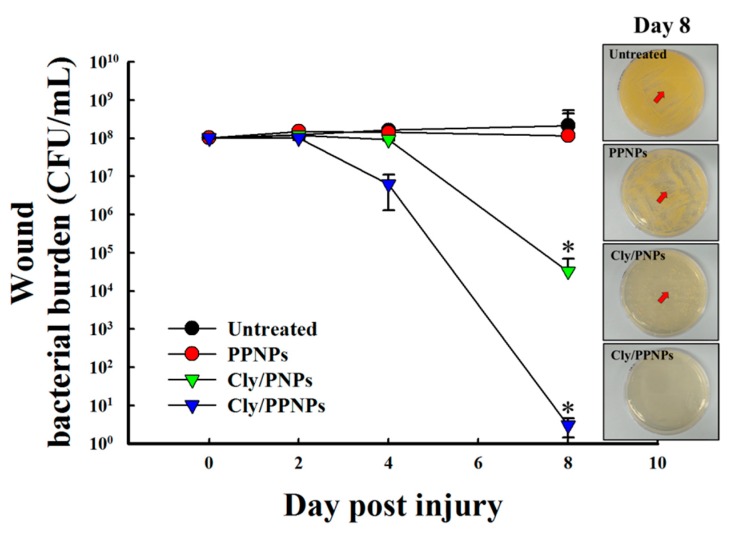
Viable counts of bacteria on wounds. Wounds were swabbed, and bacterial burden was examined. Inset shows the bacterial growth after plating of swab samples on tryptic soy broth (TSB) agar at day 8 post injury.

Bacterial infection of wounds is known to delay wound healing. Therefore, we evaluated the bacterial burden in wound by viable cell counts. As shown in Figure 7, in the untreated and PPNPs groups, the bacterial burden on wounds did not decrease until day 8 post injury. In contrast, the Cly/PNPs-treated groups experienced a >3-log reduction of bacterial viability (~99.9% of killing) at day 8 post injury. The Cly/PPNPs-treated groups showed a higher level of reduction of bacterial burden i.e., >1-log reduction (~90% killing) at day 4 post injury, followed by >6-log reduction (99.9999% of killing) at day 8 post injury. The inset figures show MRSA bacteria plated out from wound swabs taken on day 8 post injury. The untreated and PPNP groups yielded a higher number of MRSA colonies compared with the Cly/PNP and Cly/PPNP groups with no bacterial colonies found with the latter.

## 4. Discussion

In this study, our aim was to develop a clindamycin delivery system designed to target the MRSA cell wall to improve antibiotic efficacy in treating MRSA-infected wounds. PEI was used as positively charged donor polymer, and PLGA was used as the NP-forming polymer and drug depot to enable the sustained release of the drug. PLGA itself is FDA-approved copolymer that can be used for diversity of biomedical applications such as implants, sutures, bone regeneration, prosthetic devices, sutures and NPs [39]. In particular, PLGA-based drug delivery system has been considered a promising carriers for wound healing activity because several reasons, e.g., (1) PLGA?itself a wound healing agent, (2) PLGA carriers enable the protection of the encapsulated drugs, improve bioavailability, solubility, and stability, and effectively deliver drugs with a reduced dose, (3) the controlled and sustained release of therapeutic drugs from PLGA carriers promote efficient wound treatments, (4) combined wound healing effects of PLGA (lactate) and a loaded drug can alleviate the healing process of wounds [40]. In the past decade, antimicrobial NPs and antibiotic incorporated in polymeric NPs have been proven to be effective in treating infectious diseases and possess anti-MRSA activity [28]. However, to further enhance therapeutic outcomes with minimal side effects, targeted delivery of antibiotics to bacteria would be ideal. In this study, we developed positively charged Cly/PPNPs and the negatively charged Cly/PNPs were used as counterpart particles. We chose NP-bacterial targeting due to the fact that the composition of bacteria cell walls possesses negatively charged enable electrostatic interaction with positive charged NPs. All of NPs were characterized and the result showed that the particle sizes of PPNPs, Cly/PNPs, and Cly/PPNPs did not differ, demonstrating that the addition of PEI or clindamycin does not alter particle size or size distribution. The positive charge of PPNPs and Cly/PPNPs is attributed to the orientation of the protonated primary amines of PEI located on the surface of NPs. The positive charge clindamycin-loaded NPs were successfully developed and are a key factor in enabling bacterial cell wall-NP adhesion for the treatment of MRSA-infected wounds.

Before evaluating the effectiveness of the positively charged NPs in treating MRSA-infected wounds, we first evaluated the in vitro drug release at physiological conditions (PBS, pH 7.4, 37 °C). The drug release mechanism from PLGA NPs is diffusion- and degradation-controlled [41]. Clindamycin was released from Cly/PNPs and Cly/PPNPs in a biphasic pattern (Figure 2C). This release pattern explains the efficiency of the antibacterial activity of clindamycin, i.e., the initial fast release kills bacteria and prevents bacterial growth, and the sustained release contributes to maintaining an adequate concentration of clindamycin at the site of action. Sustained release of clindamycin from NPs also lower the frequency of drug administration and dose, resulting in improving patient compliance and prevent the emergence of drug resistance [42].

Since the positive charge of NPs is a key factor in determining the adhesion of NPs to the bacterial cell wall. We hypothesized that the positive charge Cly/PPNPs would facilitate the electrostatic interaction of the NPs to the negatively charged bacterial cell wall, thereby increasing the antibacterial activity. Bacterial cell wall components such as teichoic acid, lipoteichoic acids, peptidoglycan, pyruvylated polysaccharides, teichuronic acids, succinylated lipogylicans, and lipopolysachharides contribute a negative charge of the bacteria surface [43]. Mechanism of action of clindamycin is by inhibiting protein synthesis; the location of target action is inside the cell wall of bacteria. Therefore, the attachment of positively charged NPs to the bacterial wall is required to enable the penetration of antimicrobial agents into the cell [44]. To test this hypothesis, we studied the binding of NPs to the bacterial surface using confocal microscopy. Negatively charged Cly/PNPs were used as means to compare the performance of the positively charged Cly/PPNPs. Figure 3 shows the proof to our hypothesis that Cly/PPNPs bind strongly to the bacterial cell wall even after being detached through sonication, whereas Cly/PNPs have no ability to bind to the bacterial cell wall. These results also confirm that the association between the NPs and the bacteria is an electrostatic interaction between opposite charges.

After confirming the adhesion of NPs to bacteria, we next tested the effect of the adhesion on antibacterial activity. We first tested the antibacterial activity of the PPNPs, since the polycationic nature of PEI is known to exert antibacterial activity [45,46]. However, MRSA was not susceptible to the PPNPs, regardless of the NP concentrations, implying that the majority of the PEI in the PPNPs is located in the interior of the PLGA NPs, exposing only a small portion on the surface of the NPs. In contrast, Cly/PNPs and Cly/PPNPs killed MRSA in a concentration- and time-dependent manner (Figure 4A), indicating that bacteria viability can be killed either by short incubation times at high concentrations or by long incubation times at lower concentrations. The efficacy of bacterial cell wall-NP adhesion is shown in Figure 4B, where washing completely removed unattached NPs, leaving only the attached form to carry out antibacterial activity. These results demonstrated that Cly/PPNPs have greater efficacy than clindamycin alone. Based on the results, we propose that Cly/PPNPs improve antibacterial activity of clindamycin via the adhesion of Cly/PPNPs to MRSA which enables a high local drug concentrations and more efficient penetration of clindamycin through the bacterial membrane. The underlying mechanism of NP antibiotic carrier with the surface charge approach has been validated in other studies, such as cationic Eudragit/PLGA NPs, chitosan NPs, antimicrobial peptide, and cationic peptide-based NPs [47,48,49,50]. Because of the promising therapeutic application of Cly/PPNPs for the treatment of MRSA-infected wounds, we evaluated their cytotoxicity, along with clindamycin base, PPNPs, and Cly/PNPs on healthy mammalian fibroblast cells (L929). Mammalian fibroblasts cells accommodate a proper model for in vitro cytotoxicity studies because they play a significant role in wound healing, epithelial-mesenchymal interaction, and the development of the extracellular matrix [51]. As illustrated in Figure 5, all NPs exert no significant cytotoxicity to L929 fibroblast cells regardless of their concentration, indicating that surface-exposed PEI and clindamycin released from NPs are harmless to healthy fibroblasts. Thus, clindamycin and the polymers used in this study are safe for topical application, particularly in situations that require the absence of toxicity, as in the case of newborn tissues.

Based on the evidence shown above, Cly/PPNPs are effective against MRSA infection by suppressing bacterial growth while exerting no cytotoxic effects in mammalian tissue, making it a high priority candidate for development as a wound healing agent. Full-thickness wounds in mice were inoculated with MRSA, and the infection was allowed to develop for 2 days. Due to the elasticity of the mouse back skin, the tension around wound was reduced, thus can activate wound contraction [52]. To minimize the effect of wound contraction on wound healing, Tegaderm^®^ was used as a skin fixation method. The method is widely used and the result showed that adhesive polyvinyl chloride film such as Tegaderm^®^ can be used to fix saggy skin and to strengthen the skin tension [53]. Then the wounds were treated with the NPs every 2 days; this interval was chosen based on the clindamycin release profiles. It is worth to note that although there are several studies have been reported that lactase, the byproduct of PLGA degradation can stimulates angiogenesis and accelerate the wound healing [54], these effects were not significant in our current study as we used MRSA-infected wounds as wound models. Bacterial colonization of wounds interrupts the wound healing process by forming structures that are impenetrable to phagocytic cells and resistant to antibiotics. For this reason, the removal of the bacterial burden on the wound bed is required to prevent severe local and systemic infection and enhance wound healing. Using untreated and PPNPs groups as controls, we found that the Cly/PPNPs were able to reduce the bacterial burden and accelerate wound healing activities (i.e wound closure and re-epithelialization) to remarkable levels compared with untreated, PPNPs, and Cly/PNPs groups (Figure 6A,B). Taken together, these observations suggest that the positive charged Cly/PPNPs improve the antibacterial efficacy of clindamycin via NP–bacterial interactions, ultimately resulting reduced bacterial burden and accelerated wound healing.

The progress of wound healing was also observed by histologically via H&E staining of MRSA-infected wounds. Figure 6C shows untreated and PPNPs-treated wounds with ulceration, edema, and abundant mononuclear inflammatory cells with inflammation infiltration deep through the dermal layer. The Cly/PNPs promoted some progress in wound healing, but intact skin was unable to fully develop underneath the highly visible scab. The structure of the skin remained unhealthy, with sign of mononuclear inflammatory cells and neovascularization. In contrast, the Cly/PPNPs-treated groups showed increased numbers of fibroblast-like cells and decreased number of mononuclear inflammatory cells; healed skin structures appearing like normal, healthy epidermis were observed. Accelerated wound healing after the eradication of bacterial burden on the wound would be partially due to the effect of lactate, the metabolic by-product of PLGA in signaling collagen synthesis and wound repair [55,56].

Taken together, the positive charged clindamycin releasing polymeric NPs (Cly/PPNPs) propose several therapeutic benefits. First, clindamycin-loaded NPs can be engineered for bacterial-targeted delivery using biodegradable and biocompatible PLGA polymer which possesses the flexibility to fine-tune its surface properties [26]. Second, the adhesion of Cly/PPNPs to the bacterial cell wall enable high local drug concentrations and thus improve the antibacterial efficacy of clindamycin [28]. Third, based on the fact that Cly/PPNPs have a greater antibacterial efficacy than their constituent antibiotic (clindamycin) alone, Cly/PPNPs would be of great interest in various biomedical applications such as bacterial biofilm-infected wound therapy, MRSA-infected diabetic wounds therapy, and concomitant delivery of multiple antimicrobial agents for synergistic effects.

## 5. Conclusions

In this study, bacteria-targeted, clindamycin-loaded polymeric NPs were successfully developed. The adhesion of NP to the bacterial cell wall can improve the efficacy of clindamycin for the treatment of MRSA-infected wounds. The clindamycin-loaded PLGA NPs sustained the release of clindamycin over 2 days. The Cly/PPNPs had higher levels of adhesion to the negatively charged bacterial cell wall and were more efficient in killing bacteria compared with Cly/PNPs. Moreover, the application of the Cly/PPNPs to MRSA-infected wounds is beneficial to wound healing by decreasing wound bacterial burden and accelerating the reduction in the size of wounds. Therefore, the development of positively charged of clindamycin-releasing polymeric NPs with the ability to target the bacterial cell wall is a promising approach to enhance wound healing and treat various skin infections.

## Figures and Tables

**Table 1:** Characterization of nanoparticles (NPs). DLS, dynamic light scattering; SEM, scanning electron microscopy; PDI, polydispersity index.

NPs	Drug Loading (% *w*/*w*)	Size (nm)		PDI	Zeta Potential (mV)
		DLS	SEM		
PPNPs	Not determined	193 ± 38	184 ± 36	0.15	+17 ± 0.50
Cly/PNPs	1.43 ± 0.46	132 ± 41	141 ± 43	0.14	−16 ± 0.20
Cly/PPNPs	1.31 ± 0.26	126 ± 33	147 ± 37	0.10	+13 ± 0.60

Values are expressed as mean averages ± SD of three different batch of particles. PPNPs: PLGA-PEI NPs.
